# A scoring model based on ferroptosis genes for prognosis and immunotherapy response prediction and tumor microenvironment evaluation in liver hepatocellular carcinoma

**DOI:** 10.18632/aging.203721

**Published:** 2021-11-28

**Authors:** Lei Gao, Juan Xue, Xiaomin Liu, Lei Cao, Ruifang Wang, Liangliang Lei

**Affiliations:** 1 Department of Gastroenterology, The First Affiliated Hospital, And College of Clinical Medicine of Henan University of Science and Technology, Luoyang, China; 2 Department of Clinical Laboratory, The First Affiliated Hospital, And College of Clinical Medicine of Henan University of Science and Technology, Luoyang, China; 3 Department of Gastrointestinal Surgery, The First Affiliated Hospital, And College of Clinical Medicine of Henan University of Science and Technology, Luoyang, China

**Keywords:** liver hepatocellular carcinoma, ferroptosis, model, prognosis, immunotherapy

## Abstract

Ferroptosis is a type of iron-dependent programmed cell death. Ferroptosis inducers have been shown to have a great potential for cancer therapy. We aimed to generate a risk scoring model based on ferroptosis-related genes (FRGs) and validate its predictive performances in overall survival (OS) prediction and immunotherapy efficacy evaluation in liver hepatocellular carcinoma (LIHC). Differential and Univariate Cox regression analyses were applied to analyze RNA-seq data of LIHC samples from TCGA and GEO databases to identify prognosis-related ferroptosis genes. Patients were assigned to three clusters (Ferrclusters A, B, and C) based on the cluster analysis of prognostic ferroptosis genes. The principal component analysis (PCA) was performed to build a risk scoring model based on differentially expressed FRGs. Survival analysis revealed that Ferrcluster B LIHC patients had a lower OS rate alongside more severe immune cell infiltration versus Ferrcluster A and C patients; moreover, the LIHC patients in high-ferrscore group had significantly lower survival than the low-ferrscore group. Compared to low-ferrscore patients, *Programmed cell death 1 (PD-1)* mRNA expression significantly increased, and either PD-1 or PD-1 plus CTLA4 (cytotoxic T-lymphocyte associated protein 4) inhibitors showed unsatisfactory efficacy in high-ferrscore patients. Our study demonstrates the implication of FRGs in prognosis prediction and evaluation of immunotherapy efficacy in LIHC patients.

## INTRODUCTION

LIHC, the most common primary malignant tumor of liver, is one of the most threatening malignancies to human life, with the rising morbidity and the growing mortality [1, 2]. Accordingly, it continues to be the leading cause of cancer death worldwide [3]. The LIHC occurrence and development is recognized as a highly complex process involving multiple systems (e.g., gene mutation, chromosome change, gene copy number variation, and interaction of multiple signal pathways) [4]. Thus, the molecular mechanism and characteristics in the occurrence of LIHC should be clarified, and biomarkers capable of predicting the diagnosis and prognosis of LIHC patients and guiding LIHC treatment should be studied.

Ferroptosis refers to a novel cell death mode that is determined by iron and reactive oxygen species (ROS) [5, 6]. In general, it is accompanied by considerable iron accumulation and lipid peroxidation during ferroptosis [7, 8]. Ferroptosis is primarily characterized by an obvious mitochondrial contraction in the morphology, a significant increase in the membrane density, and the reduction or the disappearance of mitochondrial cristae, which is inconsistent with autophagy, apoptosis and other programmed death patterns [9–11]. As reported in existing studies, ferroptosis is associated with various diseases including malignant tumor [5, 12]. According to recent studies, ferroptosis is critical to kill cancer cells and inhibit tumor growth, and cisplatin and other chemotherapy drugs combined with ferroptosis inducer Erastin exert synergistic effects on cancer treatments [10]. For the mentioned reasons, targeted ferroptosis may be a novel tumor treatment method.

Tumor microenvironment (TME) refers to the growth and survival of a tumor or cancer stem cell, which consists of the surrounding immune cells, blood vessels, extracellular matrix, lymphocytes, etc [13]. Cells in tumor microenvironment are involved in various immune reactions and activities of tumors. For instance, macrophages are capable of promoting tumor cells to escape into the circulatory system, and it can also inhibit anti-tumor immunity [14]. Fibroblasts can allow cancer cells to migrate from the primary site to the blood to cause systemic metastasis of tumors [15]. Thus, tumor microenvironment critically impacts the occurrence and development of cancer, immune escape and immunotherapy response, which can cause a range of biological behaviors to change [16, 17]. As reported in existing studies, cancer cells can activate different immune checkpoint inhibitors (immune checkpoint inhibitors, ICIs). Among immune checkpoint inhibitors, the PD-1/PD-L1 and CTLA4 inhibitors exhibit high efficacy. However, immune checkpoint inhibitors can only benefit a small number of patients, and low immune response rates and immune-related adverse reactions remain in some patients [18, 19]. However, since immune response acts as a complex process, biomarkers or models to predict the efficacy of ICIs should be capable of distinguishing effective and ineffective patients. Currently, numerous studies primarily examined biomarkers (e.g., immune cell infiltration, PD-L1 overexpression, copy number change and somatic mutation) [20]. As impacted by the individual differences and tumor heterogeneity, the accuracy of biomarker prediction will change. In this study, combined with the LIHC data in the TCGA and the GEO, the immune cell infiltration, ferroptosis genes mutation and copy number change of the TME in LIHC were comprehensively analyzed, and the ferroptosis scoring model was built to quantify the expression level of immune checkpoint inhibitors (e.g., the *PD-1*, *PD-L1* and *CTLA4*) in LIHC patients, and then the prognosis, tumor microenvironment and application prospect of the immunotherapy in LIHC patients were analyzed and predicted.

## RESULTS

### Somatic mutation and copy number variation (CNV) of LIHC ferroptosis genes

**Figure 1:**
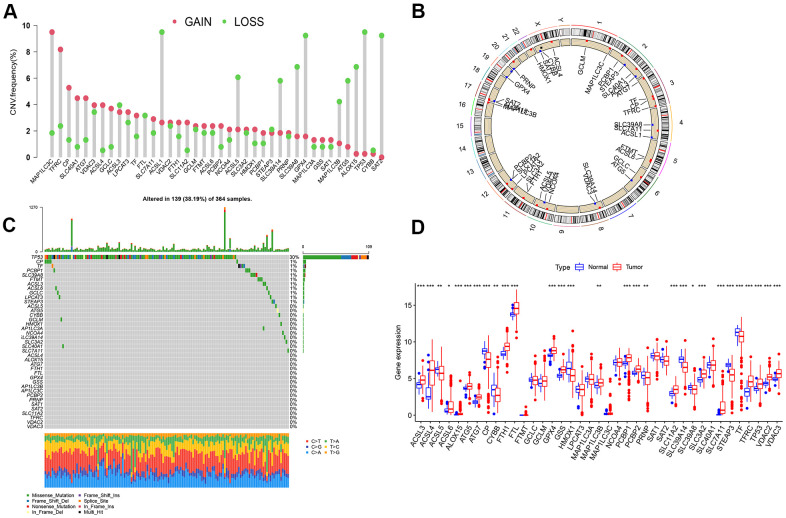
**Analysis on ferroptosis genes variation and its expression in LIHC.** (**A**) Frequency of copy number change of 40 ferroptosis genes in LIHC; the height of the column represents the frequency of change; the green dots represent the frequency of deletions; red dots represent copy number increase frequency. (**B**) 23 pairs of human chromosomal ferroptosis gene copy number change position. Red dot represents that the sample with the increased copy number was larger than that with the deletion copy number, while blue dot represents the opposite. (**C**) Somatic cell mutation of ferroptosis genes in 364 LIHC samples was detected, and 139 (38.19%) had mutations. The maximal mutation frequency was *TP53*. The upper bar graph presents TMB, and the number on the right represents the mutation frequency of the respective regulator. The bar graph on the right illustrates the proportion of the respective mutation type, and the bar graph below represents mutation transformation. (**D**) Expression of Ferroptosis genes in normal and LIHC samples (***P < 0.001, **P < 0.01, *P < 0.05).

Clinical characteristics of included LIHC cases from the TCGA database is shown in Table 1. The CNV and somatic mutation frequency of 40 ferroptosis genes in LIHC was first analyzed. According to the results, ferroptosis genes in LIHC generally increased in copy number. However, the frequency of missing copy number for *ACSL1*, *ACSL5*, *SLC39A14*, *SLA39A8*, *MAP1LC3B*, *ATG5*, *ALOX15*, *TP53*, *CYBB* and *SAT2* exceeded that of copy number increase (Figure 1A). Figure 1B presents the location of ferroptosis genes copy number changes on human chromosomes. For ferroptosis genes, 139 (38.19%) of the 364 samples had somatic mutations, and the maximal mutation frequency was *TP53*, which mutated in 109 samples (30%) (Figure 1C). The expression level of ferroptosis gene in normal and LIHC tissues was examined. As revealed from the results, most ferroptosis gene expression was up-regulated in LIHC tissues, while *ACSL5, CP, HMOX1, PRNP, SLC39A14, SLC39A8,* and *TF* expressions in normal tissue were significantly higher than those in tumor tissues (Figure 1D).

**Table 1:** Clinical characteristics of included LIHC cases from the TCGA database.

**TCGA cohort**	**Case size (%)**
Age (years)	
Mean±SD	59.57±13.33
Sex	
Female	121(32.18)
Male	255(67.82)
Grade	
G1	55(14.63)
G2	180(47.87)
G3	123(32.71)
G4	13(3.46)
Unknow	5(1.33)
Stage	
I	175(46.54)
II	87(23.14)
III	85(22.61)
IV	5(1.33)
Unknow	24(6.38)
T stage	
T1	185(49.20)
T2	95(25.27)
T3	80(21.28)
T4	13(3.46)
Unknow	3(0.80)
N stage	
N0	256(68.09)
N1	4(1.06)
Unknow	116(30.85)
M stage	
M0	271(72.07)
M1	4(1.06)
Unknow	101(26.86)

### Ferroptosis genes subtype, biological function analysis and immune cell infiltration analysis among different subtype

**Figure 2:**
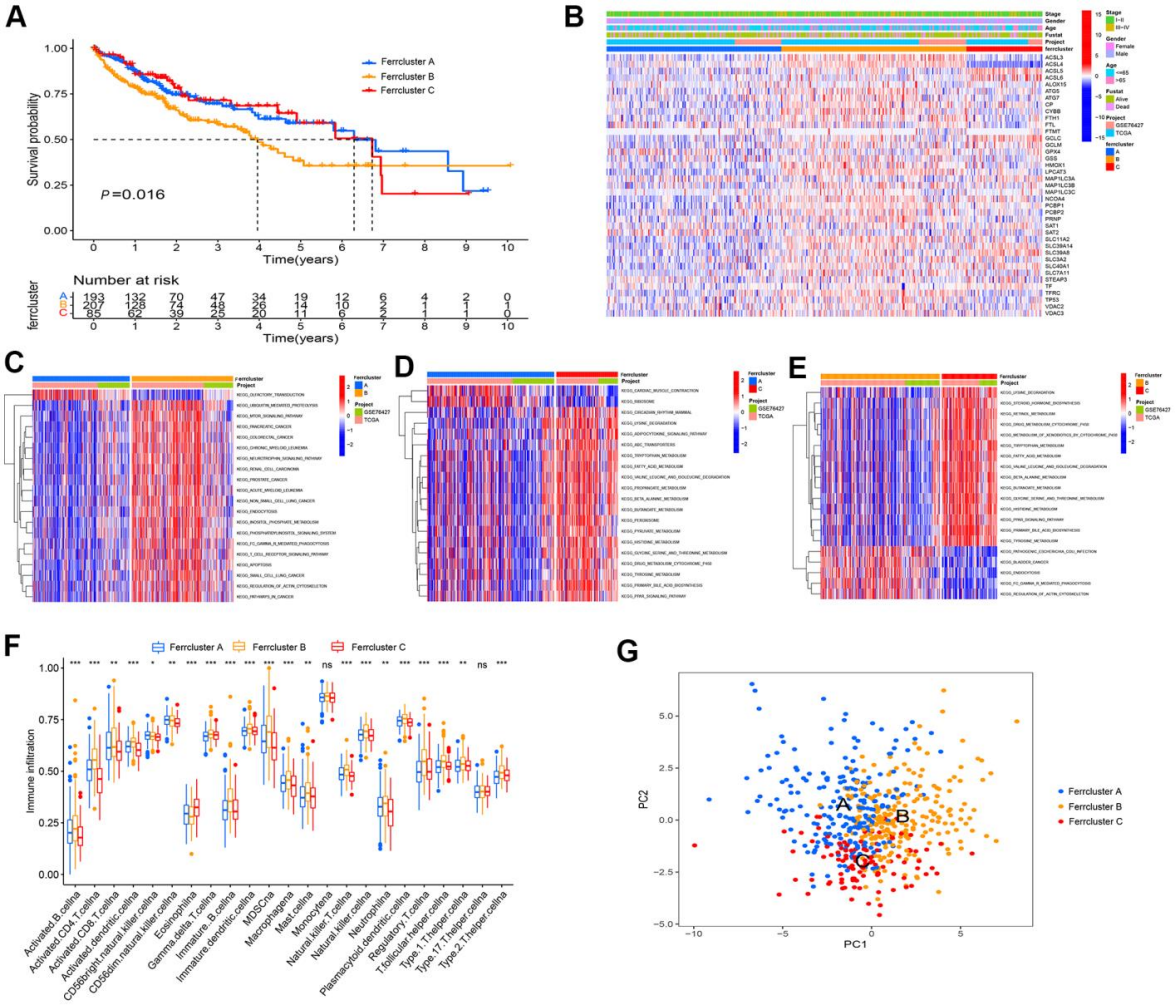
**Ferroptosis genes subtype, pathway enrichment analysis and TME infiltration.** (**A**) GEO and TCGA cohort were combined, and unsupervised cluster analysis was conducted on 538 LIHC samples, i.e., 213 cases with Ferrcluster A, 222 cases with Ferrcluster B and 103 cases with Ferrcluster C. Kaplan-Meier curve showed statistically significant difference in the survival among the three types (P=0. 016). (**B**) Thermogram results showed the expressions of different clinical traits in the three types. (**C**–**E**) The GSVA enrichment analysis reported the activation of pathways among different subtypes. The heat maps illustrate the mentioned biological processes; red represents the activation pathway, and blue represents the inhibitory pathway; (**C**) Ferrcluster A *vs.* Ferrcluster B; (**D**) Ferrcluster A *vs.* Ferrcluster C; (**E**) Ferrcluster C *vs.* Ferrcluster B. (**F**) The infiltration of immune cells in TME in 3 subtypes, the upper and lower ends of the box indicate the quartile range of the value. The lines in the box denote the median value (***P < 0.001, **P < 0.01, *P < 0.05). (**G**) Principal component analysis indicated significant differences in the three types.

The TMB of the combined LIHC samples is shown in Supplementary Table 1. To verify whether 40 ferroptosis genes are correlated with the prognosis of LIHC, it was found that 29 ferroptosis genes including *TP53* could act as a prognostic biomarker of LIHC by univariate COX analysis (Supplementary Table 2). 538 LIHC samples were clustered according to the expression level of ferroptosis genes, which were divided into Ferrcluster A to C. Among them, there were 213 cases in cluster A, 222 cases in cluster B, and 103 LIHC patients in cluster C (Supplementary Table 3). The survival analysis indicated a difference in the survival among the three types of LIHC patients (*P*=0. 016). The overall survival rate of patients with Ferrcluster A and C reached over that of Ferrcluster B. According to the results of the heat map, most ferroptosis genes were slightly expressed in Ferrcluster A and highly expressed in Ferrcluster B. Moreover, there were more patients with advanced stage and death in Ferrcluster B, which further explained why the overall survival rate of s Ferrcluster B was lower than that of Ferrcluster A and C (Figure 2A, 2B).

As indicated from the GSVA enrichment analysis, the Ferrcluster A was mainly enriched in “Olfactory transduction” and “cardiac music contraction”. The Ferrcluster B was largely enriched in the “mTOR signaling pathway” and “neurotrophin signaling pathway”. Ferrcluster C was mainly enriched in “adipokine signaling pathway”, “tyrosine metabolism” and “PPAR signaling pathway” (Figure 2C–2E). TME immune cell infiltration analysis showed that Ferrcluster B had very rich immune cells (e.g., B cell, CD4 ^+^ T cell, immune B Cell, natural killer T cell, MDSC, macrophagena as well as master cell), and the Ferrcluster C was significantly lower than the other two types of B cells and T cells. (Figure 2F). Subsequently, the principal component analysis of three different subtypes of ferroptosis reported a difference between the three different subtypes, i.e., ferroptosis-related gene could successfully distinguish the LIHC sample (Figure 2G).

### Differential gene screening and prognosis gene subtype

**Figure 3:**
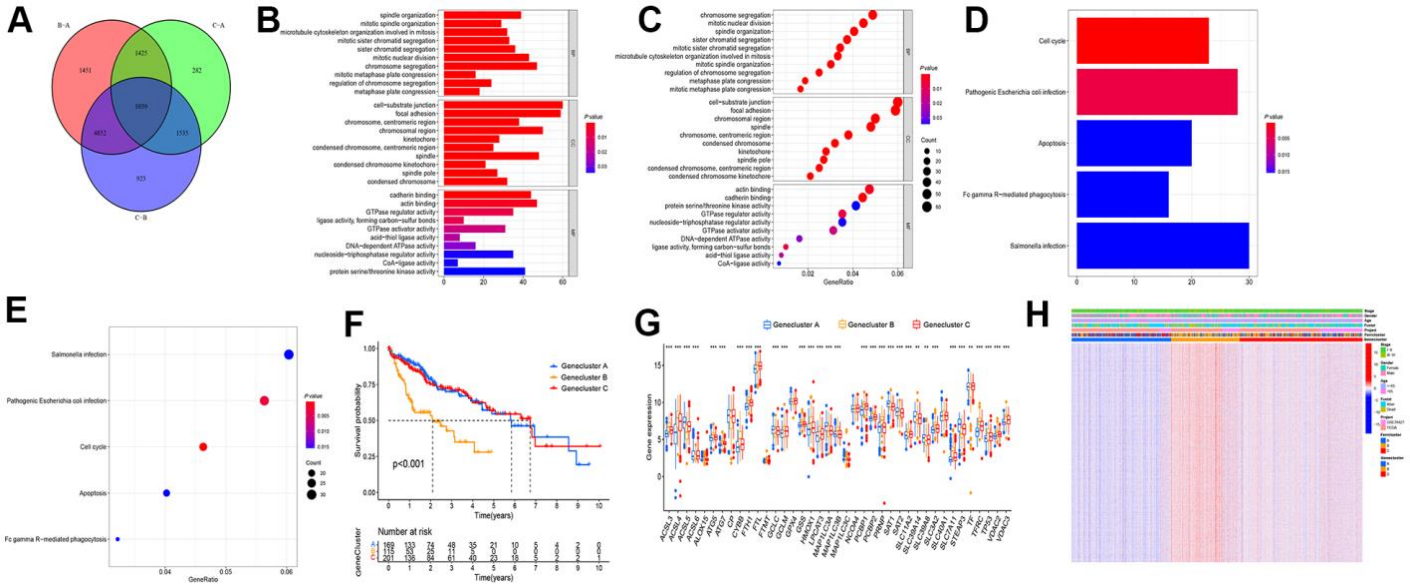
**Screening differential gene among the subtypes of differential genes.** (**A**) Wayne diagram was adopted to screen the difference genes in the three types, and 1039 genes were obtained after taking the intersection. (**B**–**E**) GO and KEGG enrichment analysis were performed on the intersection gene. (**F**) Cluster analysis was conducted on the intersection gene, and the patients fell to ferroptosis Genecluster A-C, with Genecluster A, B, and C as 169, 115, and 201 patients, respectively. (**G**) Expressions of ferroptosis genes in three gene clusters. (**H**) Thermogram showed the expression of clinical features among the three gene clusters. (***P < 0.001, **P < 0.01, *P < 0.05).

To study the potential biological behavior among the three types in depth, the filter condition was set as regulated *P* value < 0.01. On the whole, 1039 differential genes were obtained in three subtypes of LIHC (Figure 3A and Supplementary Table 4). The differential genes were subjected to GO and KEGG enrichment analysis using clusterProfiler (Figure 3B–3E). Subsequently, univariate COX analysis was conducted for the prognostic analysis of the 1039 differential genes to screen the prognostic genes. Moreover, the cluster analysis of prognostic genes was conducted to classify LIHC patients into three gene subtypes. To be specific, 169 LIHC patients were ferroptosis Genecluster A, 115 patients were ferroptosis Genecluster B, and 201 patients were ferroptosis Genecluster C (Supplementary Table 5). The survival analysis reported a difference in the survival among the three gene subtypes (*P* < 0.001), and the overall survival rate of Genecluster A and Genecluster C reached over that of Genecluster B (Figure 3F). The box chart presents the distribution of ferroptosis gene in three gene subtypes (Figure 3G). As suggested from the heat map results, LIHC patients in Genecluster B were mainly patients with stage III − IV, and most patients died of LIHC. As opposed to the mentioned, LIHC patients in Genecluster A and C were mainly patients with stage I − II, and the survival state was primarily survival patients (Figure 3H).

### Construction of ferroptosis scoring model

**Figure 4:**
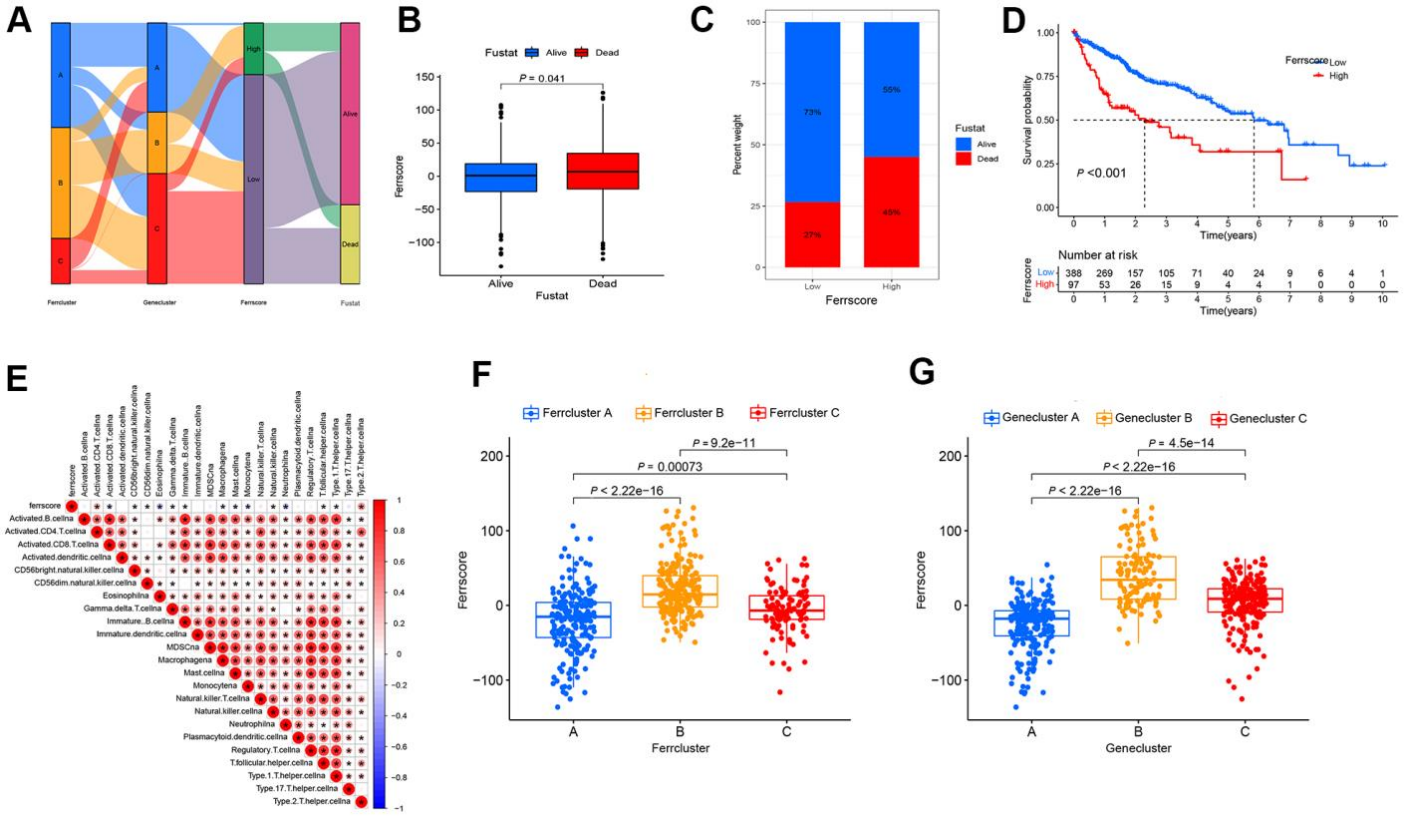
**The built ferroptosis scoring model.** (**A**) Sanky diagram is used to show the attribute changes of individual liver cancer patients, showing the relationship between ferrcluster, genecluster, ferrscore and survival status. (**B**) Box chart shows ferrscore of dead and surviving patients, and the difference showed statistical significance (*P* < 0.05). (**C**) The survival status of patients in the high and low ferrscore groups, red represents death and blue represents survival. (**D**) Kaplan-Meier curve was used to analyze the survival of patients with high and low ferrscore liver cancer (*P* < 0.001). (**E**) The Spearman correlation analysis was conducted to examine the relationship between ferrscore and immune infiltrating cells. (**F**) The Kruskal-Wallis test was carried out to compare the statistical differences in the three types of Ferrcluster A-C (*P* < 0.001). (**G**) The Kruskal-Wallis test was performed to compare the statistical differences among the three gene subtype ferroptosis Genecluster A to C (*P* < 0.001).

With a view to the individual heterogeneity and complexity of LIHC patients, we built a scoring model based on the mentioned prognosis-related ferroptosis genes, so as to accurately predict the prognosis and immunotherapy prospect of a single LIHC patient, quantify the ferrscore of LIHC patients, and facilitate individualized treatment (Supplementary Table 6). Sankey diagram presents the attribute variations of individual patients (Figure 4A). In accordance with cut-off values, 97 LIHC patients were classified as high-ferrscore groups. 388 patients were in the low-ferrscore group (Supplementary Table 7). As indicated from the histogram results, the ferrscore of patients who died of LIHC was larger than that of patients who survived LIHC (*P* = 0. 041) (Figure 4B). The survival patients took up 73% of the low-ferrscore group; 55% of the patients survived in the high-ferrscore group. The survival analysis revealed the differences in the survival between high- and low-ferrscore groups, and the high-ferrscore group had significantly lower survival than the low-ferrscore group (*P* < 0.001) (Figure 4C, 4D). For the correlation between the ferrscore and immune cells, ferrscore was negatively correlated with CD8^+^T cell, Eosinophil, Macrophagena, Mast. Cell, Monocyte, Neutrophilia and other immune cells, and it was positively correlated with CD4^+^T cell, Type. 2. T. helper. cells (Figure 4E). According to the results of the Kruskal-Wallis test, the ferroptosis genes cluster and the Ferrcluster were significantly inconsistent with the ferrscore, and the medians of the Ferrcluster B and the Genecluster B demonstrated the median scores of the Ferrcluster A and the Genecluster B as the maximal, while those of the Ferrcluster A and the Genecluster B as the minimal (Figure 4F, 4G).

### Ferroptosis score and TMB analysis and somatic mutation

**Figure 5:**
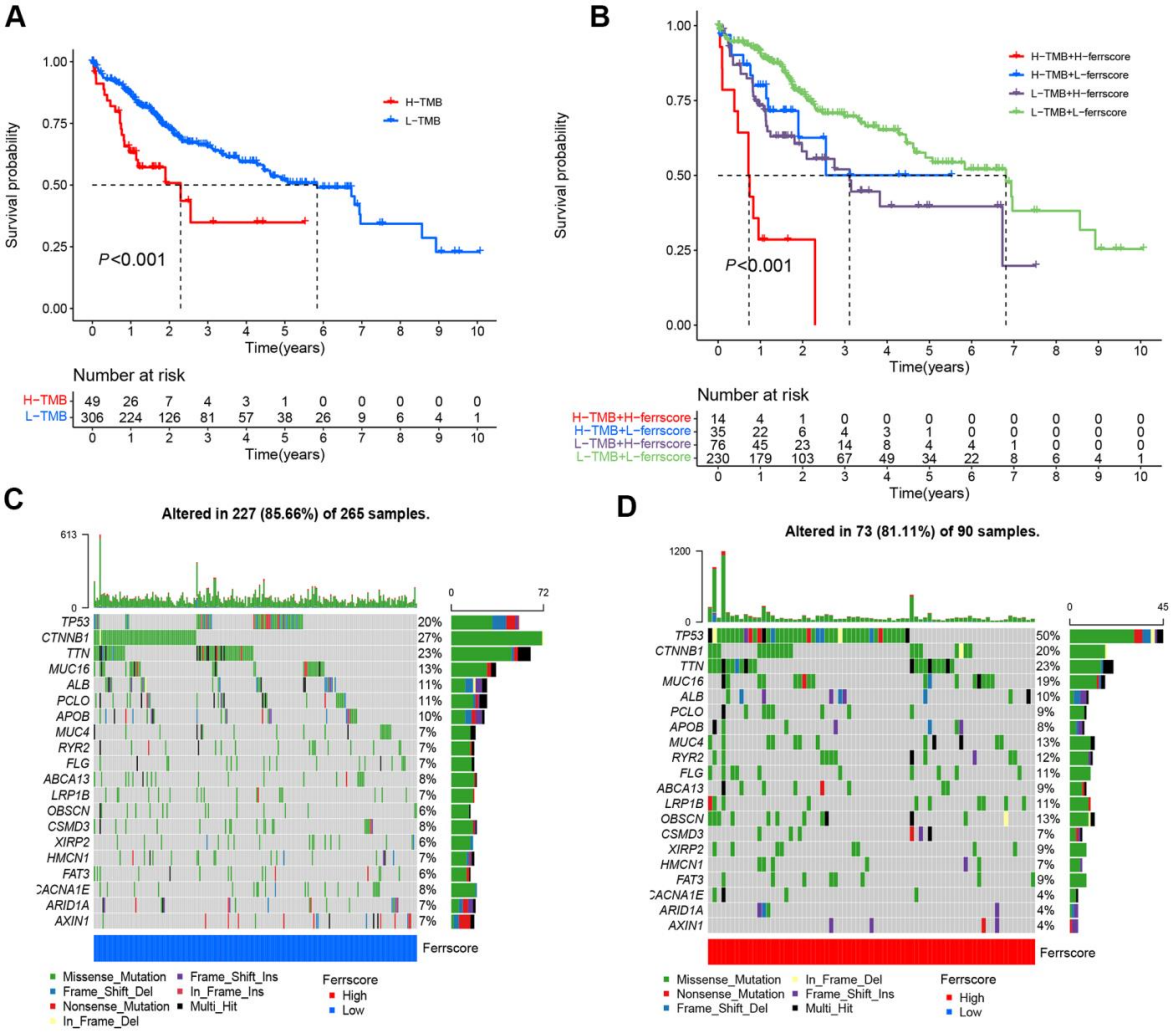
**Ferroptosis score and tumor mutation analysis.** (**A**) The Kaplan-Meier curve was adopted to analyze the survival of patients with liver cancer in the high and low ferrscore groups (*P* < 0.001). (**B**) The survival analysis of patients stratified with the ferrscore and the TMB by using the Kaplan-Meier curve (H=high; L=Low). A cascade chart of tumor somatic mutation established by patients with low ferrcore (**C**) and high ferrscore (**D**). Each column represents an individual patient. The bar chart above shows the TMB, and the number on the right represents the mutation frequency of the respective gene. The bar chart on the right presents the proportion of each mutation type.

Given the cut-off value and TMB data of LIHC patients, LIHC patients fell to the high and low mutation load groups. To be specific, 49 LIHC patients were in the high mutation load group, and 306 patients existed in the low mutation load group. The survival analysis suggested a difference in the survival between the high and low tumor load groups (*P* < 0.001). The low mutation load group achieved the better survival than the high mutation load group (Figure 5A). As indicated from the joint analysis for ferrscore and the TMB analysis, the low TMB group combined with the low-ferrscore group achieved the optimal survival, the survival of the high tumor mutation group combined with the high-ferrscore group was the worst; the difference was statistically significant (*P* < 0.001) (Figure 5B). Moreover, the “Maftools” package was adopted to analyze the somatic mutations in the high-and low-ferrscore groups in the TCGA-LIHC cohort. The relevant results are presented in the figure, and the somatic mutation rate of the low-ferrscore group reached 85.66%, of which the CTNNB1 mutation rate was the maximal (27%), mainly the missense mutation. The mutation rate in the high-ferrscore group was 81.11%, of which the *TP53* mutation rate was the maximal (50%), primarily the missense mutation (Figure 5C, 5D).

### Clinical application of ferroptosis score and analysis of immunotherapy prospect

**Figure 6:**
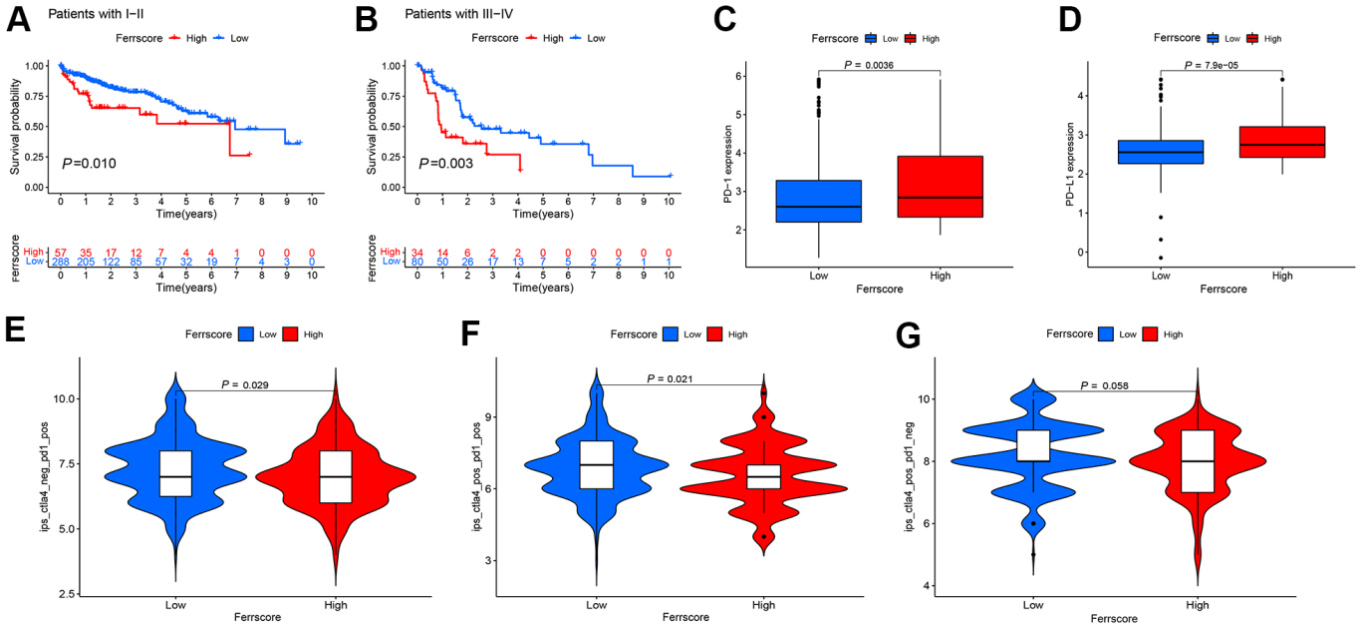
**Application of the ferroptosis scoring model in clinic and prospect of immunotherapy.** (**A**, **B**) The Kaplan-Meier curve was adopted to analyze the survival of I-IV liver cancer patients in the high and low ferrscore groups (*P* < 0.001). (**C**, **D**) The Wilcoxon test was performed to analyze the expression of *PD-1*/*PD-L1* in the high and low ferrscore groups, and the difference showed statistical significance (*P* < 0.05). (**E**, **F**) Patients with low ferrscore received the PD-1, and these patients having received the combination of the PD-1 and the CTLA4 achieved more effective results than patients with high ferrscore. In panel (**G**), no difference was reported in the CTLA4 treatment between high and low ferrscore groups (*P* = 0.058).

Whether the ferroptosis scoring model is suitable for LIHC patients with different stages was verified, and there were 345 patients with stage I – II. To be specific, 57 cases were from the high-ferrscore group, 288 patients were in the low-ferrscore group. 114 patients had stage III − IV, including 34 cases in the high-ferrscore group. As suggested from the results of the survival analysis, the difference between the high- and low-ferrscore groups was statistically significant (*P* < 0.05) (Figure 6A, 6B), and this ferroptosis scoring model was suggested to be suitable for clinically patients with stage I-IV in clinical practice. The expression of immune checkpoint inhibitor genes *PD-1/PD-L1* in the high/low-ferrscore groups was assessed. As revealed from the results, the expression of *PD-1/PD-L1* in the high-ferrscore group was significantly higher than that in the low-ferrscore group (Figure 6C, 6D). According to the immunotherapy results, the low-ferrscore group received the PD-1, and these patients having received the combination of the *PD-1* and the *CTLA4* achieved the better results than the high-ferrscore group (*P* < 0.05) (Figure 6E, 6F). The *CTLA4* treatment alone did not differ in the high/low-ferrscore groups (*P* > 0.05) (Figure 6G).

## DISCUSSION

Ferroptosis refers to an iron-dependent, non-apoptotic form of cell death. It can occur in numerous organ systems, which is associated with considerable diseases (e.g., central nervous system degenerative disorders, antiviral immune response and tumor) [7]. As the most influential anti-cancer treatment method over the past few years, it plays a role by activating the killing activity of T cells. Wang et al. [21] reported that CD8^+^T cells, classic tumor kill T cells, inhibit cystine uptake by tumor cells by down-regulating SLC3A2 and SLC7A11, as an attempt to enhance Ferroptosis-specific lipid peroxidation in tumor cells. After the anti-PD-L1 treatment with an immune checkpoint inhibitor, the ferroptosis-specific lipid peroxidation levels were significantly elevated. However, blockage of ferroptosis results in a significant decrease in the sensitivity of tumor cells to immunotherapy, showing that ferroptosis can increase the anti-tumor efficacy of the immune system [22]. In LIHC, sorafenib, the only approved first-line treatment drug for advanced LIHC, is capable of playing a cytotoxic role in LIHC by inducing ferroptosis [23]. Accordingly, ferroptosis, a biological process that can affect both the immunotherapy of LIHC and sorafenib, may significantly impact the clinical treatment of LIHC.

In the present study, the somatic mutation rates, CNV and TMB of 40 ferroptosis genes in LIHC was initially analyzed. In addition, *TP53* achieved the maximal mutation rate according to the results. *TP53* plays a dual role in tumor ferroptosis. Existing studies reported that TP53 can inhibit SLC7A11 to enhance ferroptosis. In colorectal cancer, the activity of DPP4 is blocked by transcription-independent method, so as to inhibit Ferroptosis [24, 25]. However, the effect of *TP53* in LIHC remains to be unclear. Subsequently, TCGA and GEO databases are combined. According to the expression level of ferroptosis genes, LIHC samples fell to the Ferrcluster A-C. TME immunocyte infiltration analysis indicated the significantly different characteristics of infiltrated immunocytes in the three types. The occurrence and development of cancer were affected by TME infiltration and associated with the host immune system [26]. Thus, TME components and immune system biomarkers are crucial for predicting the therapeutic efficacy and prognosis of patients [27].

As revealed from existing studies, according to the infiltration of immune cells, the immune microenvironment generally falls to three types, i.e., the immune rejection, the immunoinflammatory type as well as the immune desert type. The mentioned phenotypes prevent immune response from removing tumor cells through their own unique mechanism. In the present study, according to the difference of immune cell infiltration among the three types, Ferrcluster A may be immuno-inflammatory, which is characterized by infiltration of immune cell subtypes. The main reason is the increase of TIL. Ferrcluster B may be immune rejection. Despite the abundant infiltration of immune cells, the aggregated immune cells increased at the edge of the infiltration, and no effective infiltration was achieved. Ferrcluster C may be immune desert type, and the main reason is the lack of activation and initiation of T cells [28]. As suggested from the survival analysis, the cluster B patients achieved the significantly lower overall survival rate than the cluster A and the cluster C, which might be related to its immune rejection type. Combined with the results of GSVA analysis, the Ferrcluster B might inhibit the anti-tumor effect exerted by immune cells via the mTOR signaling pathway, which was consistent with the existing reports that mTOR, i.e., a central integration factor of intracellular and extracellular growth signals and cell metabolism, critically impacts the development and activation of immune cells [29]. Accordingly, the immune cell analysis based on the TME infiltration is capable of predicting the survival of LIHC patients clinically.

Subsequently, the potential biological behaviors among the three genotypes were analyzed by screening the differential gene of the three genotypes. the Univariate COX analysis was conducted to screen the prognosis-related differential genes, and the gene subtype analysis was conducted. The survival analysis reported the differences among the three gene types. Given the individual heterogeneity of ferroptosis genes and the heterogeneity and complexity of LIHC patients, a ferroptosis scoring model (using the ferrscore as a quantitative indicator) was built to quantitatively analyze individual LIHC patients. Consistent with the ferrcluster survival analysis, the overall survival rate of the Genecluster B was lower than that of the Genecluster A and C as well. Furthermore, the higher the ferrscore, the worse the survival would be. As the immune rejection type, the Ferrcluster B and the Genecluster B achieved the maximal scores, while as the immune inflammation type, the Ferrcluster A and the Genecluster A had the minimal scores, suggesting that based on the proposed ferroptosis scoring model, it has high clinical practicability, can analyze the type of TME infiltration in a single LIHC patient, and can also play a role in predicting the prognosis of LIHC patients.

In addition, this study revealed somatic mutations in the high and low ferrscore groups. In the high ferrscore group, the mutation rate of *TP53* was 50%, complying with the result achieved in existing studies. On the whole, *TP53* mutations were missense mutations [30, 31]. In the low-ferrscore group, however, the maximal mutation frequency was *CTNNB1* (27%). As reported from existing studies, *CTNNB1* mutations occur in nearly 19%-26% of LIHC patients, capable of activating Wnt-β-catenia signaling pathway and promoting tumor progression [32]. It has been reported that TMB (TMB) may act as a biomarker for ICIs prediction as well. The higher the TMB, the better the responsiveness to ICIs will be [33]. The present study found the minimal survival rate of patients in the group with high TMB combined with high ferrscore, and patients with low TMB and low-ferrscore achieved the maximal survival rate. In addition, the blockage of PD-1/PD-L1 inhibitory pathway could activate T cells in TME and release inflammatory cytokines and cytotoxic particles to eliminate tumor cells. PD-1/PD-L1 expression detection in tissues has been suggested as the optimal way to indicate the efficacy of PD-1/PD-L1 treatment in patients [34]. With the use of the proposed model, this study reported that the high-ferrscore group had the higher expression level of *PD-1/PD-L1*. Lastly, the efficacy of the combination therapy of PD-1 and CTLA4 in LIHC patients was assessed. Nevertheless, there are still many shortcomings in this study, including the relatively uneven distribution of sample size after cluster typing, and the prognosis of patients with high- and low-ferrscore groups still needs internal verification. In addition, the predictive efficacy of ferroptosis score in clinical immunotherapy of LIHC patients still needs to be further evaluated and verified.

## CONCLUSIONS

In brief, based on the ferroptosis scoring model built in this study, the infiltration of the TME immune cells in LIHC patients could be comprehensively assessed to determine the different immunophenotypes of LIHC patients, as well as the correlation between the ferrscore and clinicopathological characteristics of LIHC patients. Such a model can accurately predict the survival rate of LIHC patients, and it critical to assessing the sensitivity of patients to the PD-1/PD-L1 inhibitors and the efficacy of combination therapy of the PD-1 and the CTLA4 on LIHC patients. This study could present more insights into the clinical immunotherapy of LIHC and formulate novel strategies to develop LIHC drugs.

## MATERIALS AND METHODS

### Data acquisition and processing

The transcriptome data and clinical data of LIHC originated from TCGA GDC (https://portal.gdc.cancer.gov/) database. The R language (version 4.0.2) and the “Bioconductor limma” package were adopted to classify the transcriptome data, and transform the RNA sequencing data (FPKM value) to the TPM value (transcripts per kilobasemillion). With “heptocelluar carcinoma survival” as the keyword, the probe matrix file and the platform annotation file of LIHC were downloaded from GEO (Gene-Expression Omnibus, https://www.ncbi.nlm.nih.gov/geo/) database, and the matrix file should involve the survival time and survival status of the patients. GSE76427 contained 116 patients with LIHC, and the clinical data of the patients were complete and qualified to be included in the subsequent analysis here. The LIHC somatic mutation data originated from TCGA. Furthermore, the data of LIHC Copy Number Variation (CNV) originated from the UCSC xena (https://xena.ucsc.edu/) database.

### CNV and mutation analysis of ferroptosis genes

The CNV increase or deletion frequency of ferroptosis was determined, as presented in the form of histogram. Based on the “RCircos” R package, the CNV of 40 Ferroptosis gene on 23 pairs of human chromosomes was analyzed [35]. With the “maftools” R package, an investigation was conducted on the mutation frequency, mutation type and base change of the ferroptosis-related gene in the TCGA-LIHC cohort [36].

### Combination of TCGA and GEO data and ferroptosis genes subtype analysis

The TCGA-TPM data of the LIHC and the GEO data GSE76427 was merged by adopting the “sva” R packet, and the expression level of ferroptosis genes was extracted. *TP53*, which has the maximal mutation frequency, was used as gene in mutation group. The expression of ferroptosis genes in TP53 wild type and mutant type was analyzed using the “ggpubr” R package [37], and the results are illustrated in box chart. The clinical data of TCGA and GEO were combined, and the survival time and survival status were preserved. The Univariate Cox regression analysis was conducted to determine whether 40 ferroptosis genes were prognosis-related genes, where *P* < 0.05 indicates that it is related to prognosis; the Kaplan-Meier survival analysis was conducted to plot the survival curves [37]. According to the expression of ferroptosis genes, the “Conensus ClusterPlus” software package was adopted, and the clustering algorithm was used to carry out ferropism gene subtype based on 1,000 iterations and a resampling rate of 80%, with the maximum subtype of 9 [38]. The principal component analysis (PCA) was conducted to verify whether ferropism gene can separate samples with different subtypes. The Kaplan-Meier survival analysis was conducted to verify whether there are survival differences between different subtypes. By combining clinical data (e.g., the TCGA and GEO survival status, age, gender and stage), the ferropism gene subtype heat map was generated.

### Gene set variation analysis (GSVA) and immunocyte infiltration analysis

To verify whether there are functional and pathway differences in the three subtypes of LIHC, the “c2. cp. kegg. v7.4. symbols.gmt” gene set file was downloaded from the MsiDB database (http://www.gsea-msigdb.org/). The GSVA analysis was conducted by complying with the “GSEABase” and “GSVA” R package [39]. The GSVA is a non-parametrical and unsupervised method, commonly employed to estimate the variation in path and biological process activity in the samples of expression datasets [39]. The regulated *P* value < 0.01 showed the statistically significant difference. The results are illustrated in the heat map.

The SSGSEA (Single-Sample Gene-Set Enrichment Analysis) algorithm was adopted to assess the infiltration content of immune cells in different subtypes of LIHC [40]. The immune data set file of tumor invasive immune cell types originated from the studies of Charoentong, and Charoentong stored human immune cells with various subtypes (e.g., activated CD8^+^T cells, activated dendritic cells, macrophages, natural killer T cells and regulatory T cells) [41]. The SSGSEA algorithm was adopted to calculate the score of each immune cell, thereby analyzing the infiltration content of immune cells among different subtypes of LIHC [40].

### Differential gene analysis among different subtypes of ferroptosis and screening of prognosis-related genes

Based on the expressions of 40 ferroptosis genes, the filter condition was set as regulated *P* value < 0.01. With the classical Bayesian method of R language limma package, the differential genes in the three types of LIHC were analyzed. The GO and KEGG enrichment analysis were conducted to examine the main functions and pathways involved by the differential genes [42]. Given the expression file of the differential genes, the prognosis-related gene was screened through the univariate COX analysis, and *P* < 0.05 indicated the association with prognosis. The consensus clustering algorithm was adopted to define the number of gene clusters as well as their stability, Kaplan-Meier survival analysis was conducted to determine the survival differences among different subtypes.

### Construction of ferroptosis scoring model

The principal component analysis (PCA) method was conducted to build the ferroptosis scoring model, and principal component 1 and principal component 2 were selected as signature scores. Ferroptosis score (ferrscore) = ∑PC1i+PC2i (where i denotes the expression amount of prognosis-related Ferroptosis gene) [43]. The ferrscore of the respective LIHC sample was determined by the formula. To conduct the survival analysis, the SurvMiner R software package was adopted to determine the cut-off values of the respective dataset subgroup [37]. The “Surv-Cutpoint” function repeatedly testing all possible tangent points to determine the maximum rank statistic was adopted for the dichotomy of ferrcore. Subsequently, patients were classified as the high and low ferrscore groups based on the log-ranking statistics of the largest choice to reduce the computational batch effect. The “survminer” R package was adopted, and the Kaplan-Meiersurvival analysis was conducted to examine the survival differences between the high and low ferrscore groups [37]. The alluvial diagram was used to visualize the changes in individual patient attributes.

### Analysis on ferroptosis score and LIHC mutation burden

The “corrplot” R package was adopted, the filter condition was set as *P* < 0.05, and the correlation graph of ferrscore and immune cells was drawn. “ggboxplot” R package was used to analyze whether there were differences in ferrscore between ferrcluster and genecluster, and *P* value < 0.05 means that the difference showed statistical significance. According to the surv-cutpoint command of “suivminer” R package, the LIHC samples were classified as the high and low mutation load groups. Kaplan-Meier was adopted to analyze the survival difference between high and low mutation loads. The TMB and ferrScore groups were combined, and the joint survival curve was drawn. Based on the “maftools” R package, the gene mutation situation in the high and low ferrscore groups was analyzed and the oncoplot command was used to visualize the data and generate the waterfall plot [36].

### Clinical application and immunotherapy prospect of ferroptosis scoring model

The clinical application scope of ferrscore model was first assessed, so as to analyze whether ferrscore is capable of distinguishing different clinical stages of LIHC, the survival status of the LIHC, as well as whether the ferroptosis gene can successfully predict the response of LIHC patients to immune checkpoint inhibitors. The boxplot of the PD-1/PD-L1 expression in the high and low ferrscore groups was drawn. The immune score file of LIHC originated from the TCIA (https://tcia.at) database, and the difference between ferroptosis score and immunotherapy score was examined, as an attempt to analyze the efficacy of the *PD1* and the *CTLA4* and their combination treatment in patients with high and low ferrscore groups.

### Statistical analysis

All data processing complied with the R software (version 4.0. 2). The one-way ANOVA and the Kruskal-Wallis test were performed to compare the differences among three or more groups. The Kaplan-Meier method was adopted to plot the prognosis curve, and the logarithmic rank test was performed to determine the significance of the difference. The Spearman test was performed to conduct the correlation analysis. All statistical *P* values were bilateral, and *P* < 0.05 was the statistically significant difference.

## Supplementary Material

Supplementary Table 1

Supplementary Table 2

Supplementary Table 3

Supplementary Table 4

Supplementary Table 5

Supplementary Table 6

Supplementary Table 7
