# Supplementary material for: A scoring model based on ferroptosis genes for prognosis and immunotherapy response prediction and tumor microenvironment evaluation in liver hepatocellular carcinoma
Source: Aging (Albany NY). 2021 Nov 28;13(22):24866–81. doi: 10.18632/aging.203721 (PMC8660622; doi:10.18632/aging.203721)
Supplement: Supplementary Table 2 [file aging-13-203721-s002.pdf]

Supplementary Table 2. Univariate COX analysis of the prognostic value of 29 ferroptosis genes for LIHC.

id	HR	HR.95L	HR.95H	pvalue	km
ACSL3	1.46379818719	1.18789336299831	1.80378576020699	0.000349177122802298	6.93758387448895E-06
ACSL4	1.05654404085644	0.982239186128069	1.13646994136896	0.139322693114131	0.0397248933577504
ACSL5	1.00876523531185	0.897669844311402	1.13360976357001	0.883451769624826	0.369194106145713
ACSL6	0.859317802283831	0.716378713861807	1.03077753572723	0.102386564883129	0.022173784832195
ALOX15	1.3593995768274	0.934719154492843	1.97702935753058	0.108120017987151	0.112389499887817
ATG5	1.84129261352537	1.3462384019538	2.51839383255051	0.00013302279860234	1.56318904487307E-08
ATG7	1.69576753268569	1.1335310326705	2.53687587020551	0.0101745242290695	0.00102490601331551
CP	0.997348662669684	0.89492218928448	1.11149814681018	0.961701741153814	0.099278931189842
CYBB	1.09998379631822	0.954093364795836	1.26818233603539	0.189301537108621	0.000475761992478674
FTH1	1.34868711348237	1.09040647133635	1.66814575838328	0.00581657558398524	0.00106578980954442
FTL	1.20028169468967	0.992543336617312	1.45149948970196	0.0597312163266482	8.28693431587091E-06
FTMT	5.718637160666	1.09617964478107	29.8334411983006	0.0385535241149496	0.151357178355422
GCLC	1.02269618402473	0.847128825453001	1.23464985890364	0.815340763806589	0.141986763337725
GCLM	1.26506228417442	1.06479694484477	1.50299321442352	0.00749529487187364	0.0000679366870214304
GPX4	1.24615278713023	0.926086227435193	1.67683820671129	0.146232961433525	0.000561928133590461
GSS	1.32808306494579	0.981173335995722	1.7976483488576	0.0662220381964282	0.0000844619929999163
HMOX1	1.17228828016108	1.03738393305447	1.32473597095017	0.0108230432292872	0.000991684837800433
LPCAT3	1.08196203690749	0.939106460597802	1.24654860596295	0.275552793491397	0.00402031037708828
MAP1LC3A	0.891083658228384	0.773260747004526	1.02685942489336	0.111010568361656	0.0433310862897966
MAP1LC3B	1.23526410146654	0.96825997912955	1.57589638450581	0.0890587150486061	0.0000856601843057803
MAP1LC3C	0.947068471117035	0.62619055059968	1.43237340155484	0.796683837735756	0.0354307708762689
NCOA4	1.19407896250527	0.948380477475154	1.50343095683875	0.131283761217079	0.0250329041557485
PCBP1	1.11593901249658	0.928924965177179	1.34060330628998	0.241135215252762	0.00463752073142654
PCBP2	1.92543680187583	1.41364874092777	2.6225092349212	0.0000324049496553362	0.0000125267500723503
PRNP	1.16767435746238	1.00623501195887	1.35501487114911	0.0411677782875497	0.0000305036009257131
SAT1	0.911865568969191	0.716054665982726	1.16122253701167	0.454427725927531	0.018233432604589
SAT2	0.879649000195695	0.71652838042782	1.07990469698253	0.220433877232993	0.0982779752876453
SLC11A2	1.14632988016343	0.916487730383393	1.4338131876635	0.231640797775481	0.0384870692106054
SLC39A14	1.05166268738388	0.906191421918889	1.22048651232375	0.507233359320072	0.0553811538994718
SLC39A8	0.975695926391048	0.821596964594237	1.15869773356118	0.779070322910226	0.140428032819877
SLC3A2	1.24371818330628	1.00278243427249	1.54254289526808	0.0471160752786631	0.00980934143661594
SLC40A1	0.982887117348084	0.818798887805583	1.17985881495019	0.853058910133915	0.0404026921205252
SLC7A11	1.3490456715743	1.17397490595933	1.55022412724077	0.0000242597698649002	1.71137940485622E-08
STEAP3	0.956319476847638	0.841286724596093	1.08708115207359	0.494580203349751	0.137444349023113
TF	0.962473579633993	0.87093830157584	1.06362917995151	0.453167966354231	0.123248580970357
TFRC	1.12032382527174	0.953765000489936	1.31596931406244	0.166503088998337	0.00319819020799827
TP53	0.936864038591002	0.77506909954809	1.13243351762675	0.500169693151779	0.00733035655433367
VDAC2	1.56306484824683	1.14107736554308	2.14110961587789	0.00540317333945014	0.00876822404005007
VDAC3	1.20193657160917	0.972860034892673	1.48495309742164	0.088209074542794	0.00801216396042403
